# Bistability versus Bimodal Distributions in Gene Regulatory Processes from Population Balance

**DOI:** 10.1371/journal.pcbi.1002140

**Published:** 2011-08-25

**Authors:** Che-Chi Shu, Anushree Chatterjee, Gary Dunny, Wei-Shou Hu, Doraiswami Ramkrishna

**Affiliations:** 1 School of Chemical Engineering, Purdue University, Indiana, United States of America; 2 Department of Chemical Engineering and Materials Science, University of Minnesota, Minnesota, United States of America; 3 Department of Microbiology, University of Minnesota, Minnesota, United States of America; ETH Zurich, Switzerland

## Abstract

In recent times, stochastic treatments of gene regulatory processes have appeared in the literature in which a cell exposed to a signaling molecule in its environment triggers the synthesis of a specific protein through a network of intracellular reactions. The stochastic nature of this process leads to a distribution of protein levels in a population of cells as determined by a Fokker-Planck equation. Often instability occurs as a consequence of two (stable) steady state protein levels, one at the low end representing the “off” state, and the other at the high end representing the “on” state for a given concentration of the signaling molecule within a suitable range. A consequence of such bistability has been the appearance of bimodal distributions indicating two different populations, one in the “off” state and the other in the “on” state. The bimodal distribution can come about from stochastic analysis of a single cell. However, the concerted action of the population altering the extracellular concentration in the environment of individual cells and hence their behavior can only be accomplished by an appropriate population balance model which accounts for the reciprocal effects of interaction between the population and its environment. In this study, we show how to formulate a population balance model in which stochastic gene expression in individual cells is incorporated. Interestingly, the simulation of the model shows that bistability is neither sufficient nor necessary for bimodal distributions in a population. The original notion of linking bistability with bimodal distribution from single cell stochastic model is therefore only a special consequence of a population balance model.

## Author Summary

Traditionally cells in a population have been assumed to behave identically by using deterministic mathematical equations describing average cell behavior, thus ignoring its inherent randomness. A single cell stochastic model has therefore evolved in the literature to overcome this drawback. However, this single cell perspective does not account for interaction between the cell population and its environment. Since stochastic behavior leads to each cell acting differently, the cumulative impact of individual cells on their environment and consequent influence of the latter on each cell could constitute a behavior at variance. Thus in nature, cells are constantly under the influence of a highly dynamic environment which in turn is influenced by the dynamics of the cell population. A typical single cell stochastic model ignores such an interaction between the population and its environment, and uses probability distribution of a single cell to represent the entire population, which may lead to inappropriate predictions. In this study, we propose a population balance model coupled with stochastic gene regulation to demonstrate the behavior of a population in which its interactive behavior with its environment is considered. Our simulation results show that bistability is neither sufficient nor necessary for bimodal distributions in a population.

## Introduction

In the study of cell populations, with vastly improved flow cytometry, access to multivariate distribution measures of cell populations has advanced considerably, calling for a concomitant application of theory sensitive to population heterogeneity. In this regard, the population balance framework of Fredrickson et al. [1] has provided the requisite modeling machinery for the same. While this recognition generally exists in the literature, the modeling of gene regulatory processes has been at the single cell level based on it being viewed as an “average” cell. Since gene regulatory processes typically involve a small number of molecules, the reaction network is stochastic in its dynamics, a feature that is included in the single cell analysis. A further issue of importance, that of bistability, occurs when two levels of gene expression, one high and referred to as “on,” and the other low and referred to as “off” exist for a given concentration of the signaling molecule. This issue is very much a part of the stochastic modeling of the single cell [2], [3]. Several kinds of stochastic models have been developed; two of them that have been broadly used are the Stochastic Simulation Algorithm (SSA) [4], [5], and the Fokker-Planck equation or Stochastic Differential Equations (SDE) [6]–[8]. The Stochastic model certainly cures the drawback of the deterministic model which describes only the averaged behavior on large populations without realizing the fluctuating behaviors in different cells.

Bistability has been studied extensively through experiments, theoretical analysis, and numerical simulations [2], [3], [9]–[11]. A bistable system is characterized by the existence of two stable steady states. The modes relating to two stable steady states appear as a bimodal distribution of the population. The coexistence of bistability and bimodal distribution has been shown in many publications [2], [3], [9], [12]–[14].

However, almost all of the modeling works on stochastic gene regulation relate to processes at the single-cell level. The outcome of numerous simulated trajectories of single cell behavior has been interpreted as population behavior. A cell is assumed to act totally independently of other cells without regard to the fact that the signaling environment is continuously altered by the concerted action of all members of the population. That no interaction between other cells has been taken into consideration in these models could indeed lead to serious bias. The drawback of the single cell model may be overcome by applying the Population Balance approach [15]. A detailed general framework of the application of population balances to microbial populations was developed by Fredrickson et al. [1]. However, the population balance model (PBM) in the cited work and many others that followed in the literature are based on deterministic behavior of the particulate entities. Ramkrishna [15] shows how the PBM can accommodate random particulate behavior described by stochastic differential equations. In this study, we demonstrate formulating a stochastic gene regulation incorporating PBM, which is capable of tracing time evolution of the behavior of the entire cell population. A system of pheromone-induced conjugative plasmid transfer [16] contributing to the dissemination of antibiotic resistance and the virulence of *Enterococcus faecalis* infections [17], [18] has been simulated in this study as an example of the critical difference between stochastic gene regulation incorporating PBM and single-cell stochastic model.

**Figure 1:**
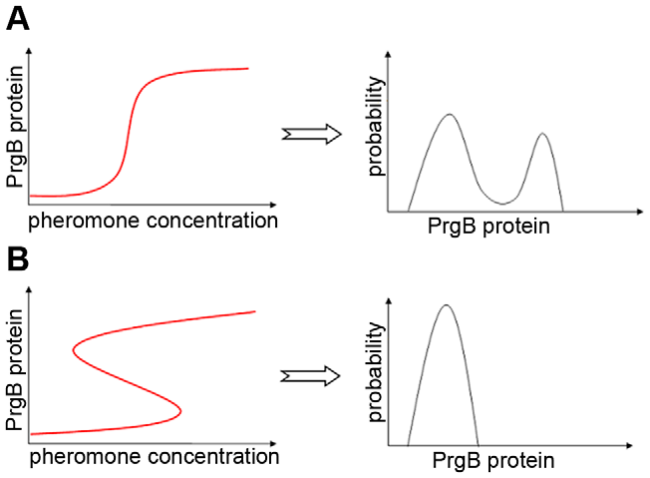
Schematic showing single cell bistability and population bimodal distribution. For cCF10 pheromone (signaling molecule) induced pCF10 conjugation system, concentration of PrgB protein indicates the level of conjugation. Our model indicates that (**A**) due to different plasmid copy number in culture of cells, bimodal population distribution can arise from cells without bistability and (**B**) due to interaction with each other, cells with bistability can abandon bimodal population distribution.

It is our objective in this paper to formulate population balance models with stochastic gene expression in single cells. Further, we explore circumstances under which bimodal distributions are observed in protein distributions; in particular we investigate the generally prevailing view in the literature that bistability and bimodality of protein distribution occur concurrently [2], [3], [9], [12]–[14]. An exception to this view appears in the work of Karmakar and Bose [19], [20], who showed that bimodal distributions can arise without bistability when the reaction time of the downstream gene regulation is short relative to the time required for change of DNA conformation. Other similar publications can also be found in literature [21]–[25]. While these cited works show bimodal distributions without bistability, it must be understood that their conclusions are based on mechanistic differences in the behavior of isolated single cells. In this study, we approach the issue of the relationship between bistability and bimodality from a rational viewpoint; i.e., to examine the nature of protein distribution from cells with and without bistability within the framework of population balances. Thus, circumstances will be investigated for Figure 1**A**, in which bimodal distributions can arise without bistability, and for Figure 1**B**, in which unimodal distributions can arise even when bistability exists.

## Methods

### The single cell deterministic (average) equations of pCF10 System

**Figure 2:**
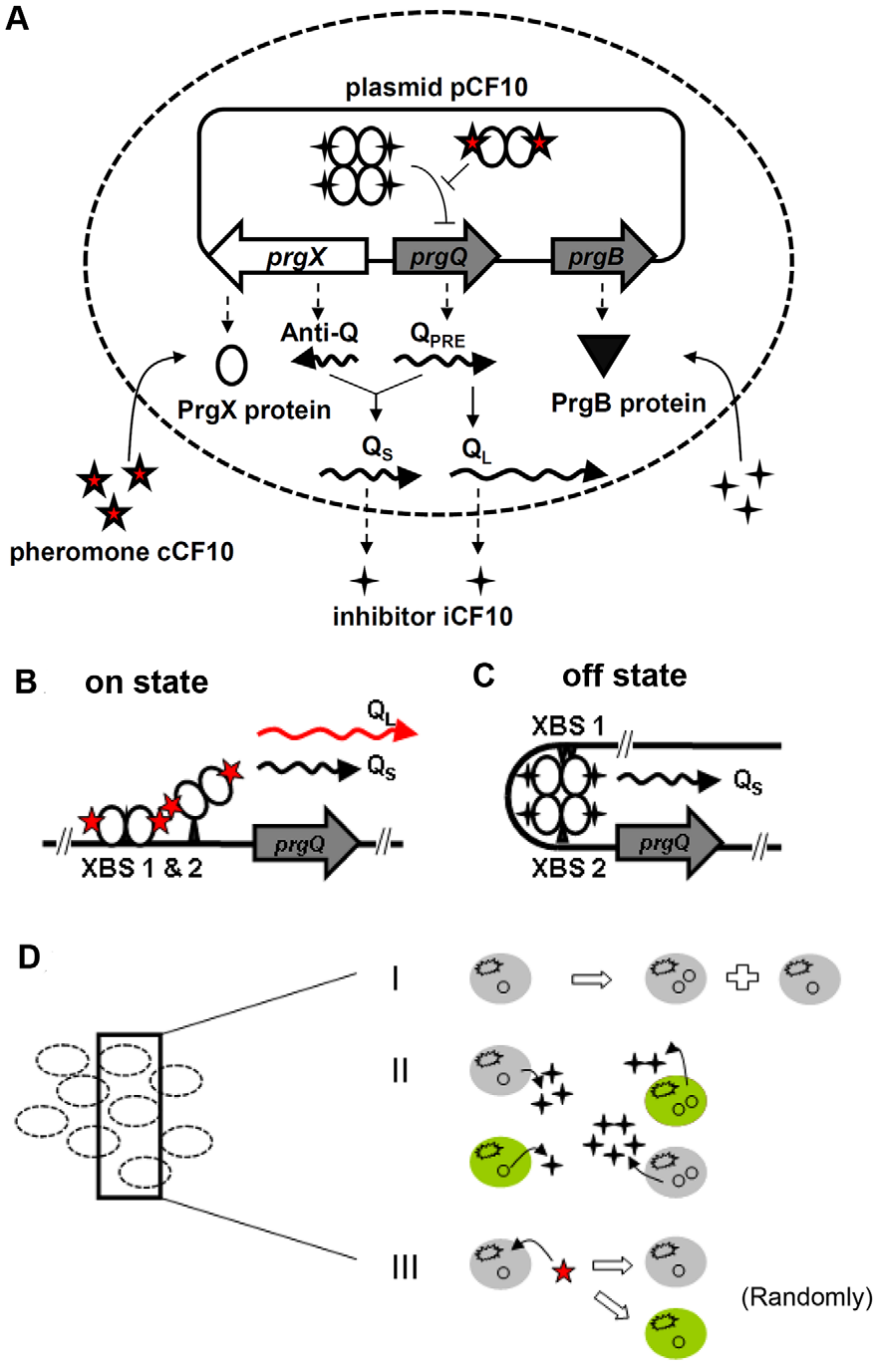
Schematic drawing of gene regulation and population balance model. (**A**) The gene reaction network of pCF10 based conjugation system. The *prgQ-prgX* gene pair regulates conjugation. While pheromone cCF10 is released by recipient cells in the extracellular environment, the inhibitor iCF10 is encoded from both Q_S_ and Q_L_ RNA, products of the *prgQ* gene. Both iCF10 and cCF10 compete for binding to PrgX protein which is assumed to exist at a constant concentration in this study. In the off state, iCF10-bound PrgX tetramers repress *prgQ* gene expression via formation of a DNA loop. Under these conditions, the nascent *prgQ* transcript Q_pre_ interacts with the non-coding antisense RNA, Anti-Q, to give rise to shorter Q_S_ RNA. In the on state PrgX-cCF10 dimers relieve *prgQ* repression to give rise to increased production of Q_pre_, which tends to titrate Anti-Q, allowing production of the longer Q_L_ RNA and consequently PrgB protein. The concentration of PrgB protein indicates the level of conjugation. (**B**) the DNA configuration of on state. (**C**) the DNA configuration of off state. (**D**) Schematic depicting the Population Balance Model (PBM) with stochastic gene regulation. Grey color indicates a cell in off state and green indicates on state. Properties of the PBM include I) Uneven distribution of plasmids to daughter cells. II) Cells with different plasmid copy number or different states act differently and influence each others. III) A cell acts random according to stochastic intracellular gene regulation.

The gene regulatory network for pCF10 based conjugation system is shown in Figure 2**A**. Under natural circumstances, pCF10 deficient recipient cells release a pheromone called cCF10 into the extracellular environment, whereas pCF10 carrying donor cells release an inhibitor molecule, iCF10 into the environment [26]. Both iCF10 and cCF10 are transported into the donor cells to interact with pCF10 DNA favoring off vs on state respectively. A pair of divergent genes *prgQ* and *prgX* present on pCF10 DNA regulates the genetic switch controlling onset of conjugation. The transcription of prgQ gene results in the formation of Q_PRE_; Q_PRE_ gives rise to two kinds of RNAs known as Q_L_ RNA and Q_s_ RNA. In the opposite direction, the *prgX* encodes the PrgX repressor and a non-coding antisense RNA called Anti-Q which may bind to Q_PRE_ [27], [28]. A Q_PRE_ bound with Anti-Q leads to shorter Q_s_ RNA. On the other hand, the final product of free Q_PRE_ is longer Q_L_ RNA. Under off conditions iCF10 bound PrgX tetramer represses *prgQ*; small amounts of Q_PRE_ are nearly all bound by overwhelming Anti-Q and result in Q_S,_ which is incapable of inducing conjugation, predominantly expressed. In the on state iCF10 bound PrgX tetramer is replaced by cCF10 bound PrgX dimer which relieves repression of *prgQ*, thus causing expression of a longer Q_L_ transcript from *prgQ* gene [29]. The Q_L_ RNA consequently results in expression of PrgB protein, an indicator for the onset of conjugation [30].

The deterministic (average) equations based on mass-action kinetics that represent the gene regulatory network in Figure 2**A** are represented below with its associated nomenclature listed in Table 1.
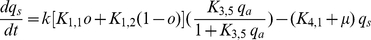
(1)

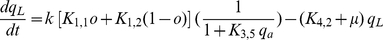
(2)

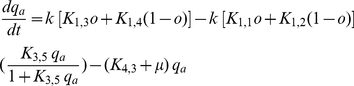
(3)

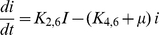
(4)

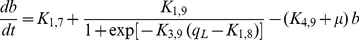
(5)Eq. (5), representing the mass balance of PrgB, treats the production rate as a sigmoid function of Q_L_ [31]. A variation considering the production rate of PrgB as a linear function of Q_L_ is contained in the modified differential equation (6) below.
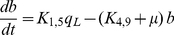
(6)


**Table 1:** Nomenclature of pCF10 system.

Notation	Name
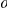	DNA of plasmid in loop form, repressed state of *prgQ*
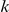	Plasmid copy number
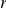	Order of DNA binding reaction, equal to 4
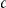	Intracellular concentration of pheromone, cCF10
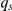	Intracellular concentration of Q_s_ mRNA
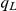	Intracellular concentration of Q_L_ mRNA
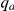	Intracellular concentration of Anti-Q RNA
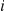	Intracellular concentration of inhibitor, iCF10
	Concentration of PrgB membrane protein
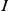	Extracellular concentration of inhibitor, iCF10
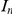	Extracellular number of inhibitor, iCF10
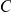	Extracellular concentration of pheromone, cCF10
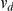	Volume per donor cell
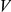	Total volume

**Figure 3:**
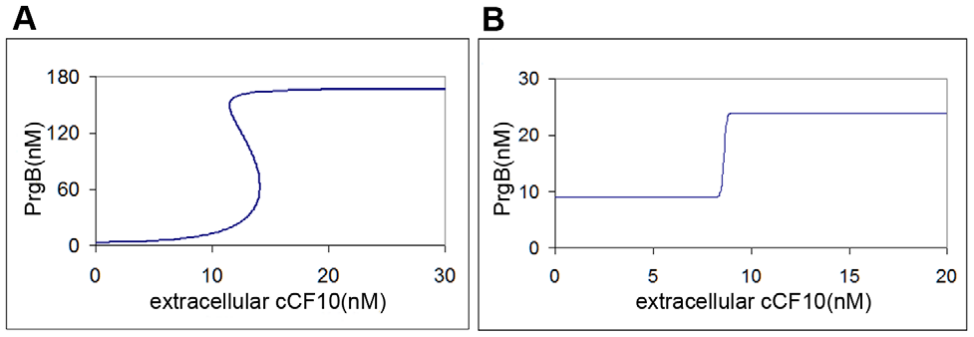
The steady-state behaviors. (**A**) System demonstrates bistability using the parameter values as shown in Table 2. (**B**) The possibility of bistability can be analytically excluded by setting the parameter value of K_4,2_ = K_4,1_ independent of other parameters. For both (**A**) and (**B**), plasmid copy number k is equal to 5.

**Figure 4:**
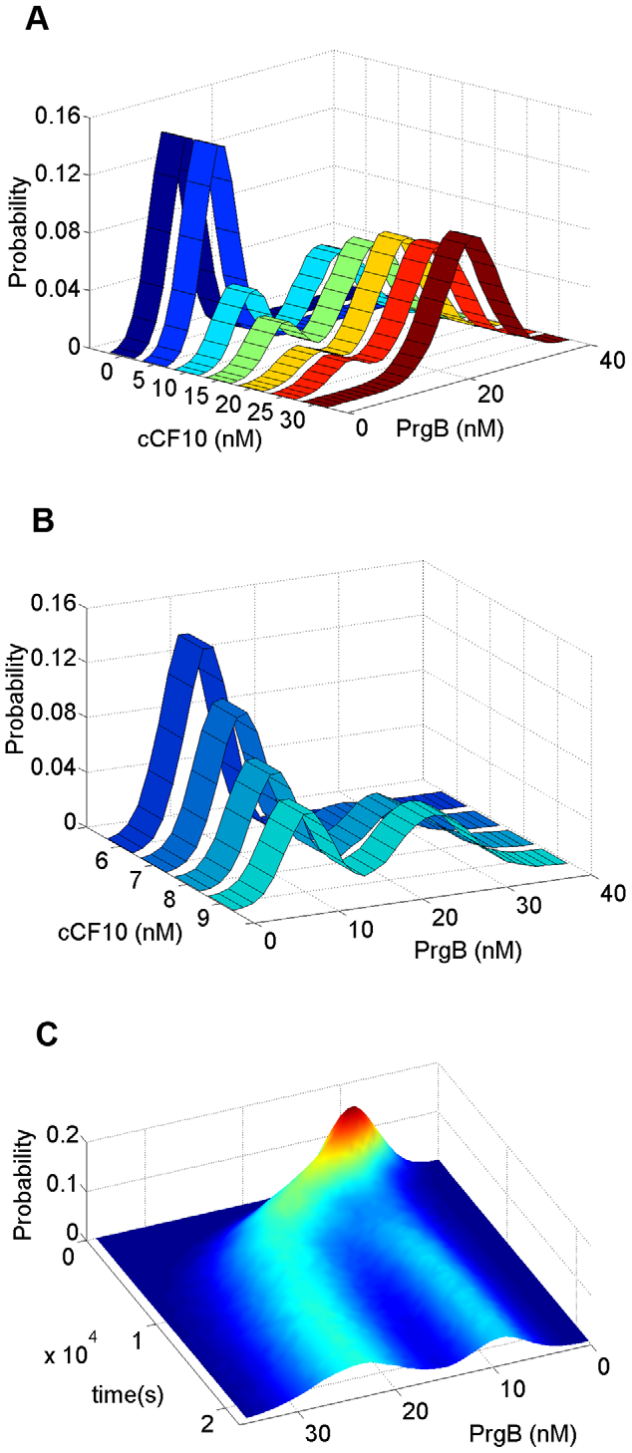
Bimodal distribution results from no bistability. The parameters used in this simulation are shown in Table 2 except for K_4,2_ = K_4,1_ = 0.001 (1/s). (**A**) The stationary distribution responding to different concentrations of extracellular pheromone, cCF10. As the concentration of pheromone increases, cell population migrates from state of low PrgB, viewed as off state, to state of high PrgB, viewed as on state. When pheromone concentration reaches 30 nM, all cells stay at on state. (**B**) The change of PrgB protein distribution for cCF10 concentration from 6 nM to 9 nM. (**C**) The dynamic behavior responding to constant concentration of pheromone, cCF10. This simulation is done with extracellular pheromone concentration maintained at 10 nM.

**Figure 5:**
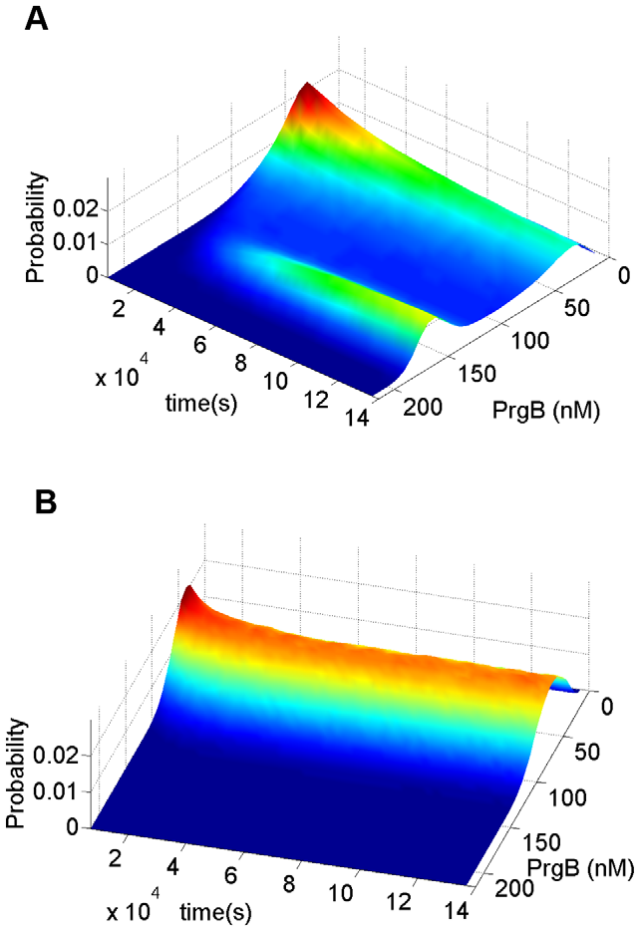
Population distribution for a system with bistability. Both (**A**) and (**B**) use the same parameters shown in Table 2. Extracellular pheromone concentration is maintained at 13.5 nM for both simulations. Only noise term of protein and peptides are taken into consideration [48], [49]. Thirty thousand cells are used. (**A**) Outcome from single cell stochastic model described in Eq.(30). Due to stochasticity, bimodal distribution comes from initial unimodal distribution. Single cell stochastic model ignores the interaction between cells. Extracellular inhibitor is used only by the donor cell secreting it. (**B**) Outcome from PBM described in Eq.(29). The extracellular inhibitor is utilized by the whole population. The simulation outcome shows that the population effect leads to unimodal distribution.

**Figure 6:**
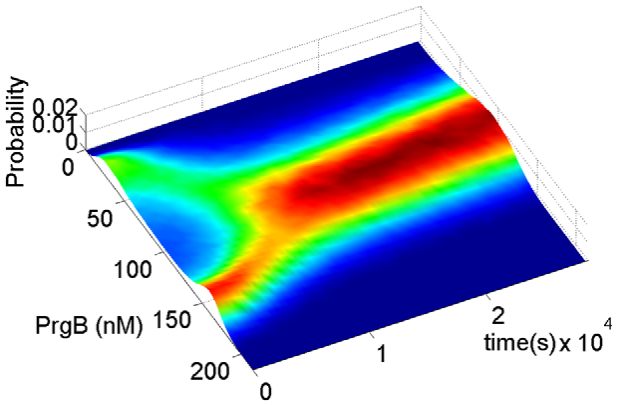
Population distribution, started with bimodal distribution, merging into a unimodal distribution due to population effect. Bimodal distribution generated from single cell stochastic model with extracellular pheromone concentration equal to 13.5 nM served as initial distribution of PBM. Simulation was done with plasmid copy number equal to 5 and parameter values listed on Table 2. Population effect of extracellular inhibitor causes two modes to merge into one.

The differential equation which remains is that expressing the mass balance of intracellular pheromone concentration given by
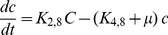
(7)In Eqs. (1) to (3), 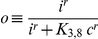, represents DNA in repressed configuration. The above equations are based on mechanisms already published in the literature [28], [30], [32]–[34]. The parameter values for the simulations, adopted from those used for a similar reaction system [35], are summarized in Table 2. RNA species Q_S_, Q_L_ and Anti-Q are described in Eqs.(1), (2) and(3), considering rate of production, degradation and dilution due to growth. The transcription rate of RNA species is modeled by a non-linear function of iCF10, cCF10 and Anti-Q to take into consideration effects of Transcriptional Interference and Antisense interaction, and are discussed in greater detail elsewhere [36]. Transport of both iCF10 and cCF10 into the donor cell is modeled as a first order reaction (Eqs. (4) and (7)). Eq. (5) considers the production rate of PrgB as a sigmoid function [31] with respect to Q_L_ and has been used to simulate Figures 3**B** and 4. Instead of sigmoid function, for reducing computational burden, Eq. (6) assumes that the expression of PrgB is linear in Q_L_ and has been used to simulate Figures 3**A**, Figure 5, Figure 6 and Text S1. The trends simulated by this deterministic model are consistent with experimental observations [17] (refer to Text S1). While the above notation for concentration serves to remind the reader of the reaction species to which it belongs, it is not convenient for their compact representation in the upcoming equations of population balance. Therefore, the intracellular concentrations are renamed as shown in Eq.(8).
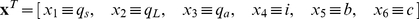
(8)


**Table 2:** Values of Parameters of pCF10 system.

Reaction constant	Name	Value	
K_1,1_	transcription rate of *prgQ*, DNA in loop form	0.0084	(nM/s)
K_1,2_	transcription rate of *prgQ*, DNA in un-loop form	0.0876	(nM/s)
K_1,3_	transcription rate of Anti-Q, DNA in loop form	0.0125	(nM/s)
K_1,4_	transcription rate of Anti-Q, DNA in un-loop form	0.0014	(nM/s)
K_1,5_	generation rate of PrgB for first order reaction	0.01	(1/s)
K_1,6_	generation rate of extracellular inhibitor, iCF10	0.005	(1/s)
K_1,7_	basic generating rate of membrane protein PrgB	0.00155	(nM/s)
K_1,8_	threshold concentration of Q_L_	12.00	(nM)
K_1,9_	rate constant of generating membrane protein PrgB	0.0031	(nM/s)
K_2,6_	importation rate of inhibitor, iCF10	0.001	(1/s)
K_2,8_	importation rate of pheromone, cCF10	2.57E-04	(1/s)
K_3,5_	equilibrium constant of Q_PRE_ and Anti-Q reaction	0.0443	(1/nM)
K_3,8_	equilibrium constant of DNA binding reaction	1.00E06	-
K_3,9_	constant of sigmoid function for Q_L_ to PrgB	12.00	(1/nM)
K_4,1_	degradation rate of Q_s_ mRNA	0.001	(1/s)
K_4,2_	degradation rate of Q_L_ mRNA	0.100	(1/s)
K_4,3_	degradation rate of Anti-Q RNA	0.0001359	(1/s)
K_4,5_	degradation rate of extracellular inhibitor, iCF10	1.00E-06	(1/s)
K_4,6_	degradation rate of intracellular inhibitor, iCF10	1.00E-06	(1/s)
K_4,8_	degradation rate of intracellular pheromone, cCF10	1.00E-06	(1/s)
K_4,9_	degradation rate of PrgB protein	1.00E-06	(1/s)
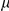	net specific growth rate of donor cells	0.0002567	(1/s)

Note that the extracellular concentration variable 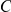 and 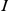 are spared from inclusion in the vector 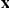. Eq. (9) provides an explanation of the different symbols in the foregoing differential Eqs. (1)–(7).
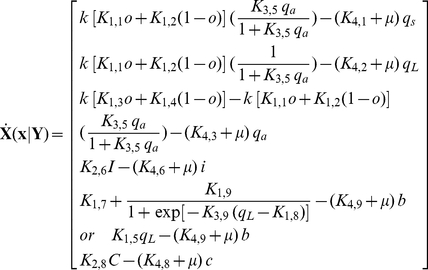
(9)where 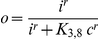, 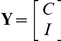


Further, the differential equation for the mass balance of 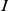 remains to be identified (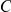 is modeled as add-in with constant concentration). Towards this end, we define 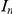 as the *number* of inhibitor molecules in the extracellular space. Assuming that the fraction of extracellular volume to total volume as constant, we identify Eq.(10) for 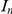 as
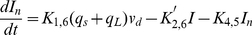
(10)where 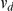 is the volume per cell. The first term on the right hand side of Eq. (10) represents the number of inhibitor molecules exiting the cell per unit time, the second their uptake rate by cells, and the third their degradation in the extracellular volume. For each cell, the uptake rate depends on the extracellular inhibitor concentration, so that the total uptake rate is proportional to the product of number of cells and the extracellular inhibitor concentration. Note that 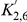 is not a constant because the uptake of inhibitor occurs by active transport and its rate depends on PrgZ protein [37]. Assuming that the uptake rate is proportional to number of the PrgZ protein and that the latter is proportional to the volume of the cell, we rewrite 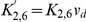 which yields 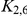 as a reasonable constant. Next, we define 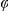 as the volume fraction of extracellular volume so that 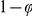 would be volume fraction of the cell, from which we have 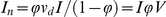
where 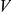 is the total volume. Substituting this into Eq. (10), using renamed variables, we obtain the differential equation for 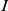 as
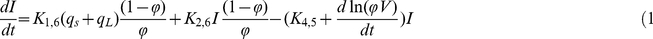
(11)The last term on the right hand side can also be represented as 
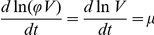
, a dilution term resulting from assuming that the volume fraction of cells remains constant as 0.5 so that an increase of cell volume by growth also results in an increase of extracellular volume to the same extent. Note that the above assumption about the extent of the extracellular volume has no influence on our conclusion. In this regard the reader is referred to the toy example where volume is modeled as constant.

### Population Balance Model (PBM) with deterministic intracellular behaviors

The effort of applying PBM on analyzing cell behaviors can be traced back to mid-twentieth century [1], [38]. More recently, the number of publications applying PBM has notably increased on analyzing complex cellular behavior (e.g. Mantzaris [39]). Thus the behavior of an entire culture of microorganisms can be simulated by PBM in the form of a multivariate population distribution. A generic formulation of PBM is presented by Ramkrishna [15]. This formulation distinguishes a vector of internal coordinates 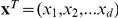 and a vector of external coordinates 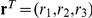; the former represents 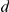 different quantities associated with the cell and the latter denotes the position vector of the cell. Cells with the same coordinates are viewed as indistinguishable. The dynamics associated with the intracellular variables through cellular processes (including gene regulation) can be described by a rate vector 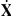 containing the deterministic reaction rates in terms of internal coordinates 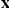. The vector 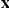 includes quantities such as cell mass, various intracellular components associated with gene expression, and so on. The vector 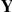 is a vector of extracellular variables influencing the intracellular processes; which may include concentrations of nutrient, signaling molecules, inhibitor and so on. The motion of cells with respect to a fixed coordinate frame may be written as Eq. (13), where the vector 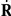 describes the velocity of the cell which may be caused by the mixing of cells (for instance, in a planktonic growth situation) or zero when imbedded in a biofilm without motion.
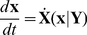
(12)

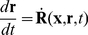
(13)where “ 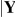 ” means “given 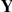
”. The notation is used to recognize the influence of extracellular variables, 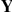 on intracellular processes. We use 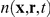 to denote the actual number density and denote its *expectation* by number density 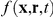 [40], for a given set of internal coordinates **x**, position coordinates **r** at a certain time 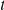. The term density here refers to number of cells per unit volume of space of internal coordinates, 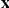 as well as that of external coordinates, 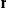. The existence of this density in physical space must be recognized even when there is no explicit dependence of cell numbers with position. The population balance equation is a state-specific balance due to various processes such as by conjugation, cell division, and so on. It may be written as
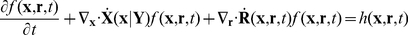
(14)The function 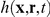 in Eq. (14) represents the net (number) rate of production of cells of state 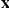 at a particular location 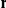 and time 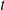. Note in particular that 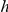, although represented as a simple function of its arguments, acquires its dependence on them through being a *functional* of the number density 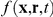. Eq. (14) is coupled with a conservation equation written for 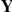, for which we define 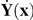 as the rate at which a cell of state 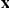 “consumes” or “contributes” to the extracellular variables in 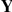. The extracellular reaction rate is described by 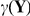. Noting that 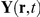 is a function of position and time, the conservation equation for 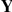 is described in Eq. (15) where 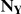 is the total flux of the various components in 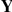 including convective as well as diffusive transport.
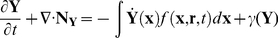
(15)The population balance model is defined by Eqs. (14) and (15), properly supplemented by initial and boundary conditions.

### PBM with stochastic intracellular behaviors

We next incorporate the intracellular stochastic behavior of gene regulation into the population balance model described in Eqs. (14) and (15). The formulation of population balance equations has been presented by Ramkrishna [15] when the internal state is a stochastic process described by the stochastic differential equation
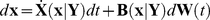
(16)where 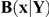 is the term that determines the magnitude of stochastic fluctuations (refer to Gillespie's Chemical Langevin equation [41]) of signal transduction reactions on the associated intracellular variables; 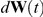, a vector, represents the increment of a standard Wiener process (during the time interval 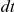). We further note that the SDE are based on Ito formulation. Also, its equivalent Fokker-Planck equation can be written as Eq.(17).
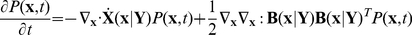
(17)where “ : ” represent double dot product so 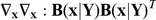 can also be written as 
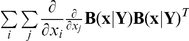
.

The population balance equation with position coordinate can be written as Eq.(18)
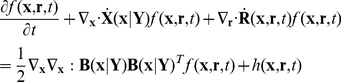
(18)which is the number balance of cells of state 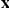 accounting for stochastic changes in internal coordinates as defined by the Ito SDE, Eq. (16). The derivation of this equation is available in Ramkrishna [15]. Next, we consider the cells to be completely (uniformly) mixed so that spatial coordinates may be eliminated. The resulting population balance equation is given by
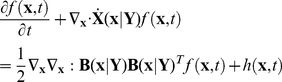
(19)Eq. (19) is coupled to a version of Eq. (15) in environmental variables 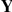 modified to account for a well-mixed system given by
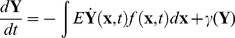
(20)where 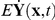 is the expected rate of consumption of extracellular variables by cells of state 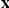. Note that 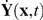 is stochastic in view of the single cell behavior being stochastic so that the cumulative contribution from a large collection of cells to the environment is deterministic given by 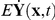. Eqs. (19) and (20) must be considered with initial and boundary conditions. Note that number density function, 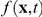, satisfies “natural boundary conditions” (i.e., vanishing of the function and its gradient at infinity). With this background, we are in a position to consider the population balance model for the system of interest, viz., conjugation of plasmid pCF10 system.

### PBM for conjugation of plasmid pCF10 system

Since the conjugative response can be influenced by the number of copies of pCF10 plasmid, we include plasmid copy number as a discrete internal coordinate for the cell in view of its effect on cell dynamics as the number of plasmids in each cell becomes important. As a result, the number density Eq. (3) is further embellished with a discrete variable representing copy number. We define the expected number density of cells 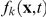, with internal state 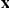 and plasmid copy number 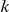, where 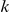 varies between 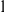 and 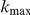. Kinetics of gene expression is denoted as 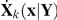 to account for the effect of plasmid copy number. Cell division rate, denoted by 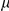, is assumed to be a constant. A more complicated situation of cell growth, which is not taken into consideration in this study, including the dependence of 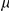 on intracellular stochastic state x can be found in Tanase-Nicola's work [42]. However, this cited work suffers from neglecting interaction between cells through population effects on the environment. The random partitioning of plasmid between daughter cells is denoted by 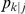 where 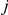 is the copy number of the dividing parent and 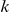 is that of the daughter cell. Plasmids replication is assumed to occur instantaneously prior to cell division. The population balance equation with stochastic intracellular behavior for this case can be written as
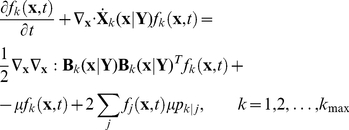
(21)Eq. (21) represents a number balance of cells of state 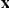 with copy number 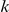. The first term on the left hand side represents the rate at which such cells accumulate, the second denotes the net flux by “convective” transport (in internal coordinate space), while the first term on the right hand side represents the mean fluctuation due to random effects, the second the loss of cells by division and the last term the gain of cells of copy number 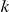 by division of other cells. The factor 2 accounts for doubling of the population by binary division. The intracellular variables of parent and daughter cells are considered to be same. The extracellular components include only pheromone and inhibitor. We do not concern ourselves with the resistance transfer process in this paper as our focus is only on the expression of the protein PrgB in the donor cells. We enunciate three further model assumptions: (i) the population density is maintained constant in the growing population by appropriate dilution; in other words, the system volume is allowed to expand suitably. This assumption is only made to provide for a true steady state in the protein level distribution. A similar assumption is also made in the single cell analysis. (ii) constant extracellular pheromone concentration, which implies that we need only consider the component 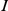 for the dynamics of the vector 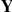, and (iii) that inhibitor is produced and secreted directly into the environment through intracellular reaction as described by Eq. (11).

The mass balance of extracellular inhibitor is adopted from Eq. (20) acknowledging any copy number dependence of the rate of consumption of extracellular variables by cells. A macroscopic mass balance for the extracellular variables, based on assumption of perfect mixing, is given by.
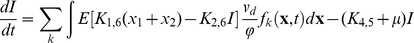
(22)In Eq. (22), we have used the renamed concentration variables 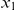 and 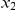 in place of 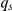 and 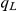 respectively. It is also worth noting that the dilution term on the second term on the right hand side comes about from the assumption that the system volume is allowed to expand to keep the population density constant. The motivation for this assumption, as pointed out earlier, is the attainment of a true steady state in the population with respect to protein levels as in the single cell analysis. A schematic illustration of PBM described by Eqs. (21) and (22) is shown in Figure 2**B**.

We restrict ourselves in this work to the case where plasmid copy number of the daughter cells is the same as that of the parent cell. In other words, if a cell of copy number 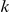 divides, just prior to division, the plasmids double in number and are equally shared by the two daughter cells. Of course more general cases are admissible in the model framework which can account for plasmid replication and uneven distribution among daughter cells. The specific assumptions in this work will, however, suffice for demonstration of population balance modeling. The even partitioning of plasmids among daughter cells in a population of *uniform* copy number distribution is described in Eq. (23).
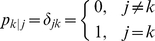
(23)Using Eq. (23) converts the population balance Eq. (21) into Eq. (24) shown below with 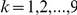, which yields an average copy number of 5 close to experimental observation.
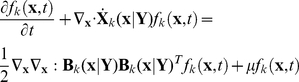
(24)The 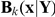 of Eq. (24) is identified below
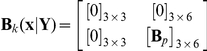
where 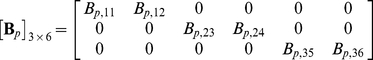


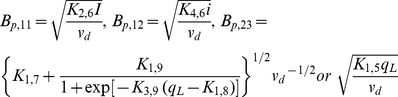


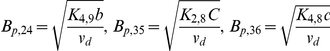
and the 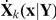 is exactly the same as 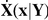 which is identified in Eq.(9). The reason for using index 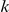 is to clearly state that PBM account for population heterogeneity of plasmid copy number. Note that Eq. (24) should be coupled with Eq. (22). The overall expected number density, denoted 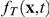, can be obtained in Eq. (25)
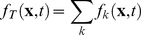
(25)


### The solution method for PBM

The difficulty involved in solving PBM with stochastic intracellular behaviors comes from the natural boundary condition (i.e., vanishing of the function and its gradient at infinity). To handle this problem, we transform the population balance equation (24) into a Fokker-Planck equation using the transformation shown in Eq. (13) to obtain Eq. (14). The integral over 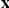 coordinates is unity because 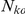 represents the initial number density of cells with copy number 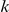.
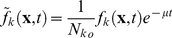
(26)

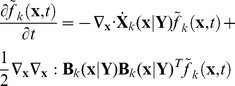
(27)Eq. (27) is a Fokker-Planck type differential equation whose solution represents the probability distribution for the stochastic process defined by the Ito SDE (refer to Eq. (16)). Thus the sample-pathwise solution to Ito SDE will provide an alternative route to calculate expectations of all quantities associated with the stochastic process 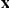, including the quantity 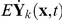, for substitution into Eq. (22). We solved Ito SDE relating to Eq. (27) by using the Euler algorithm [43]. The computation proceeds in a stepwise manner for each discrete interval to keep abreast of environmental variables.

## Results

We first compare the single cell model approach with that of population balances, delineating the differences between them. The single cell approach would use Ito SDE with variations including extracellular variables, i.e., the inhibitor in the present context, (as the inducer molecule concentration is assumed to be held constant). The secretion of inhibitor to the exterior and subsequent transport back into the cell will enter the model as a singular experience of the cell in question. Thus the environmental inhibitor concentration will display dynamics as a result of interaction with the single cell. On the other hand, in the population balance model, stochastic behavior of numerous individual cells will provide different secretions of the inhibitor to the environment which, because of mixing effects, would change the environment of all cells in the population. This will undoubtedly produce different dynamics of the system. To further elucidate the difference between the two approaches, we note that the environmental inhibitor 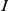 in the population balance model changes as a result of the cumulative (expected) exchange with the population whereas the single cell model will account only for the (stochastic) exchange rate with the cell's environment.

### Bimodal distribution from plasmid unevenly distributing to daughter cells

Although bistability is featured by Eqs. (1)–(10) for the range of parameter values shown in Table 2 (Figure 3**A**), bistability can be (analytically) excluded by forcing parameter 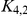, the degradation rate of Q_L_ mRNA, equal to 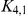, the degradation rate of Q_s_ mRNA, regardless of the values of other parameters (Figure 3**B**). The detailed derivation is shown in the Text S1.

The parameters used in this simulation are shown in Table 2, except 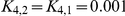 (1/s). Stochasticity is restricted to protein alone to reduce computational time. Forty-five thousand cells are used. The outcome is shown in Figure 4. Results of stationary distribution responding to different pheromone concentration are shown in Figure 4**A** and 4**B**. At zero and low pheromone concentration, all cells are at off mode. With increasing pheromone concentration, the cell population gradually migrates from mode of low PrgB, viewed as off state, to mode of high PrgB, viewed as on state. Finally, when the pheromone concentration exceeds 30 nM, all cells stay at on state. The transcription rate of various RNA species is directly proportional to the plasmid copy number. The difference between cells of various plasmid copy numbers causes population heterogeneity resulting in a distribution across the population. The results of dynamic behavior are shown in Figure 4**C**. This simulation is done for extracellular pheromone concentration equal to 10 nM. The initial distribution is obtained by simulating cells with no pheromone added. Population from initial unimodal distribution finally develops into a bimodal distribution. Figure 4 demonstrates that bistability is not necessary for a bimodal distribution and that the bimodal distribution arises directly out of population heterogeneity.

### Resolving bistability versus bimodal distribution

If there is no plasmid copy number distribution, the population balance model can then be written as:
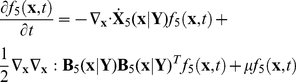
(28)

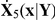
 and 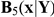 indicate Eq. (28) describing system with plasmid copy number equal to 5.

With the solution method described in Methods we convert the population balance Eq. (28) into
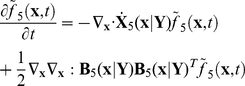
(29)Notice that above equation should be coupled with environmental equation of extracellular inhibitor Eq.(22).

The single cell stochastic model may be written as
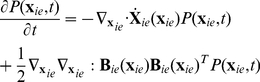
(30)Where index 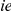 indicates both intracellular and extracellular variables are involved. For reasons that have already been elucidated earlier, Eqs. (29) and (30) are not the same. In Eq. (29), the vector 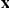 is different from the vector 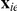 in Eq. (30) because the latter also includes the extracellular inhibitor as a stochastic variable.

The outcome of the simulation is shown in Figures 5**A** and 5**B**. While the outcome of Eq.(30) shows a bimodal distribution that of Eq.(29) shows a unimodal distribution, thus indicating the strong impact of the population on the behavior of individual cells.

In order to exclude the possibility that Figure 5**B** was a result of insufficient simulation time to develop into a bimodal distribution, a simulation was conducted with PBM using initial distribution as a bimodal distribution calculated from the single cell Fokker-Planck equation. The simulation outcome, Figure 6, shows a result consistent with Figure 5**B**. A bimodal distribution under the influence of the population effect finally merges into one mode.

### Toy example

**Figure 7:**
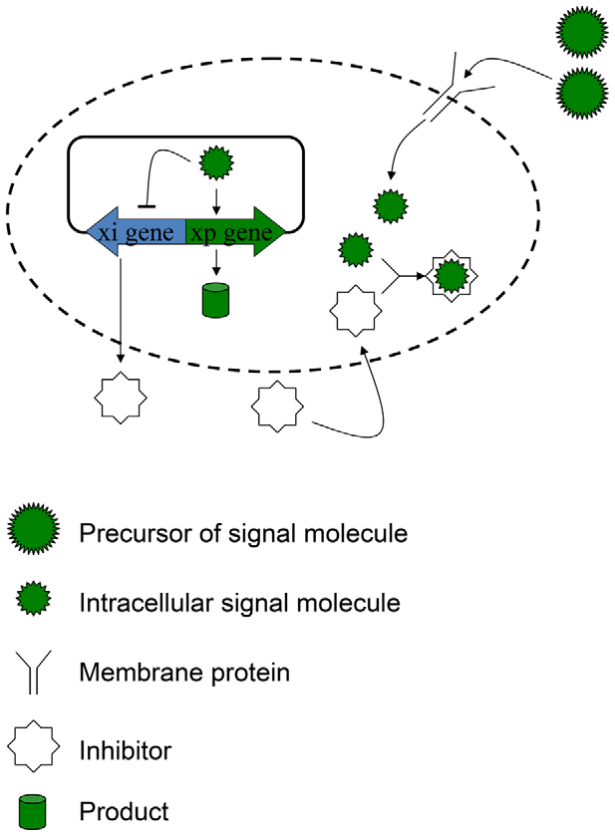
Reaction network for gene regulation in toy example. After uptake by membrane protein, the precursor of signal molecule matures into signal molecule. When signal molecule binds to DNA, it favors the expression of xp gene encoding product and represses the transcription of xi gene which encodes the inhibitor. The binding reaction of inhibitor to signal molecule is fast and irreversible so either signal molecule or inhibitor dominates.

**Figure 8:**
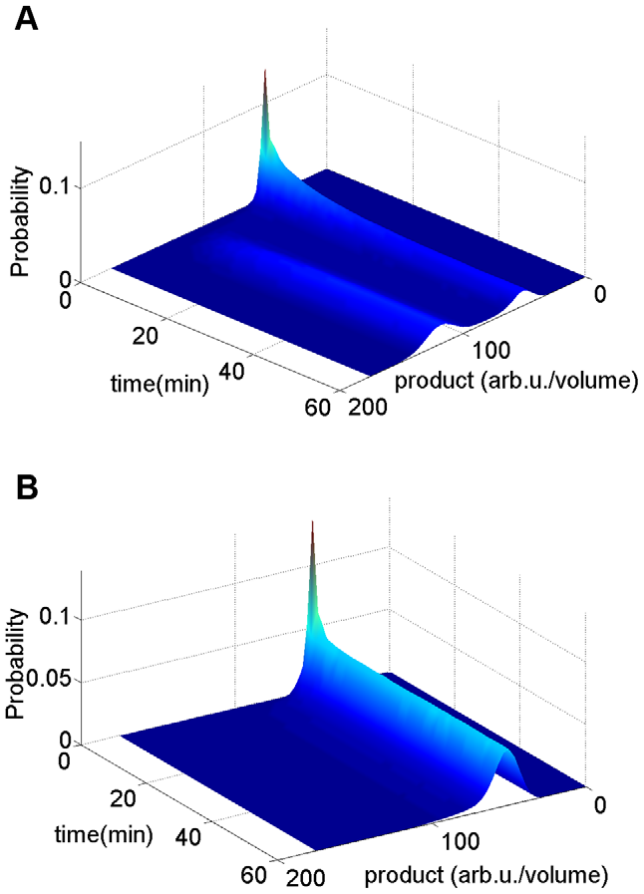
Comparison of the outcome from population balance model and single cell stochastic model in toy example. Thirty thousand cells are used with constant concentration, 10 arbitrary unit/volume, of add-in signal molecular precursor. (**A**) Bimodal distribution is observed from single cell stochastic model. (**B**) Unimodal distribution is observed from population balance model.

**Figure 9:**
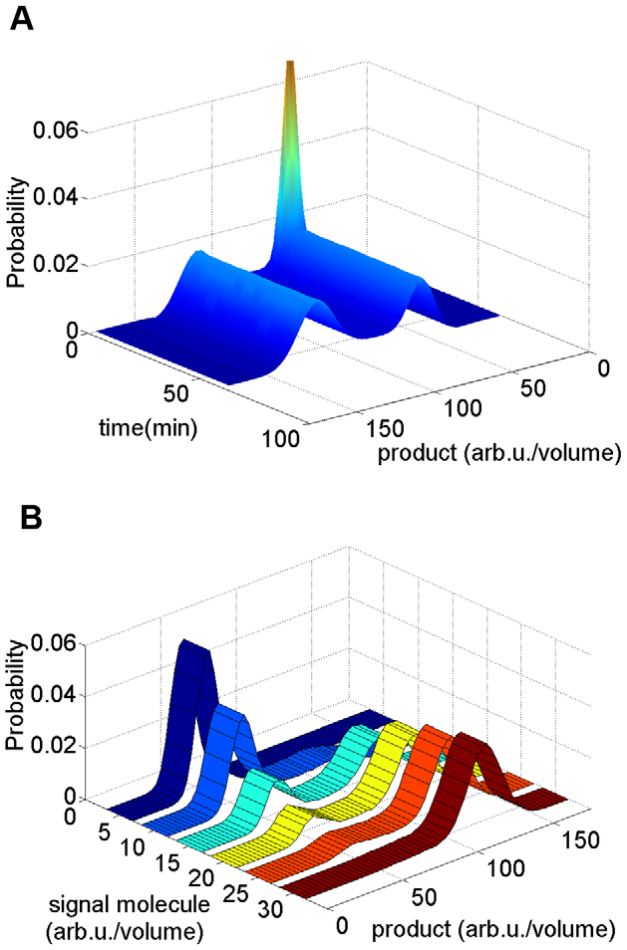
Bimodal distribution from no bistability. Thirty five thousand cells are used for simulation. (**A**) Although there is no true steady state, the effect from the increment of 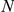 on standard deviation of the product protein distribution is small enough to consider the system as pseudo steady state. (**B**) The plot of product protein distribution with different add-in signal molecular precursor.

The purpose of this example, shown in Figure 7, is to elucidate the key elements of the more complicated model of the pCF10 System. To simplify the discussion, we use symbols to denote not only the molecular species but their concentrations. The precursor of the signal molecule, denoted as 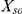, needs membrane protein, 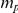, to mature into intracellular signal molecule, 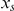. Two kinds of gene, xp gene and xi gene, encode product and inhibitor, respectively. As the signal molecule dominates, the transcription rate of xp gene is high and that of xi gene is low. On the other hand, inhibitor favors xi gene instead of xp gene by consuming signal molecule. Further, defining the intracellular concentration of inhibitor as 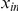, the concentration of product as 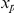, the extracellular concentration of inhibitor as 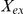, and letting 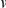 be the volume per cell, we formulate the mass balance equations for the single cell as
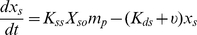
(31)

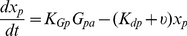
(32)

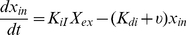
(33)

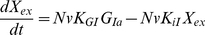
(34)where 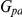 and 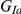 describe how DNA configurations change generation rate and are defined as
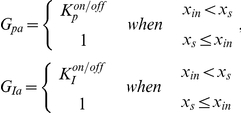
and the values of parameters are identified in Table 3. Note that 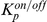 is greater than one because the signal molecule favors the transcription of xp gene and 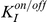 is less than one as the signal molecule prevents the transcription of xi gene. Eqs. (32) and (34), together with 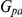 and 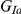 as defined above, imply that binding of intracellular inhibitor to signal molecule is an irreversible reaction with suitably large rates so that only the signal molecule or inhibitor dominates the system. The toy example is composed of seven reactions with the system at constant volume and the volume of cells negligible compared to that of the system. For intracellular variables, 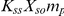 describes the generation rate of intracellular signal molecule with 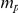 which is assumed to be controlled by a certain gene and maintained constant in each cell; 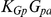 is the generation rate of product where 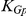 is a basic rate multiplied by a “configuration factor”, 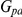; 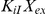 describes the uptake rate of inhibitor where 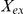 is the extracellular inhibitor concentration; 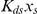, 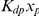 and 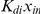 are degradation terms and 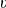 is cell growth rate. For extracellular inhibitor, the generation term includes a basic transcription rate, 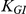, multiplied by a “configuration factor”, 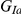. Because extracellular inhibitor is the accumulated result from all the cells, Eq.(34) further accounts for number density, 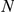. Note that although steady state exists for deterministic equation, there is no true steady state for stochastic model or stochastic gene regulation incorporating PBM. However, the effect from the increment of 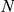 is small enough to consider the system as in pseudo steady state (refer to Figure 8 and 9).

**Table 3:** Parameters of toy example.

Reaction Constant	Name	Value	
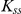	generation rate constant of intracellular signal molecule	1	(1/arb.u. min)
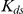	degradation rate constant of intracellular signal molecule	0.994	(1/min)
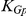	generation rate constant of product	50	(arb.u./volume min)
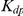	degradation rate constant of intracellular signal molecule	0.994	(1/min)
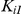	uptake rate of inhibitor	1	(1/min)
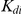	degradation rate constant of intracellular signal molecule	0.994	(1/min)
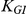	generation rate constant of extracellular inhibitor	80	(arb.u./volume min)
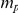	membrane protein	6	(arb.u.)
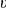	net specific growth rate of cells	0.006	(1/min)
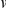	volume per cell	1E-15	(volume)
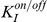	“configuration factor” of xi gene	0.5	-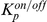	“configuration factor” of xp gene	2.4	-

The PBM with stochastic intracellular behaviors of this system is shown in Eq. (35) with environmental equation, Eq. (36). Following the method introduced in the section “The solution method for PBM”, Eq.(35) can be transferred into Eq. (37).
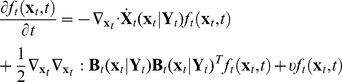
(35)

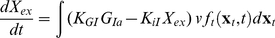
(36)

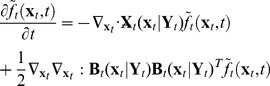
(37)where 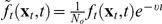 and 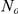 is the initial number density of cells. Each of the terms in Eq. (37) are identified below
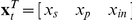


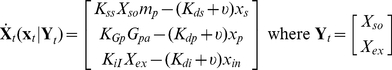


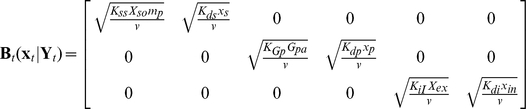
With the parameter values shown in Table 3, the deterministic Eq. (31)–(34) are featured with bistability and the outcome of single cell stochastic model, Eq.(38), shows corresponding bimodal distribution, Figure 8**A**. However, the prediction of PBM shows unimodal distribution, Figure 8**B**. The same as mentioned in pCF10 system, such qualitative difference is raised from the interaction between cells.
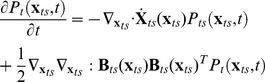
(38)Each of the terms in Eq. (38) are identified below.
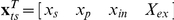


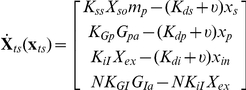


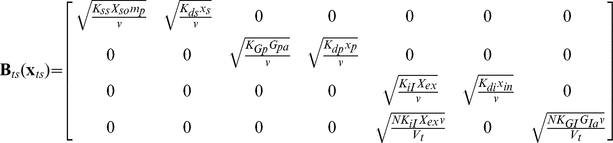
where 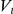 is the system volume.

Next we demonstrate bimodal distribution from no bistability. Note that the bistability can be excluded by forced 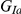 equal to one. In other words, when 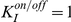, there is no bistability. Instead of 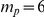, we assign 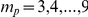 for each subpopulation and each of them starts with the same biomass concentration. The simulation outcome is shown in Figure 9. The bimodal distribution comes from population heterogeneity.

## Discussion

In this paper, we have investigated how population effect changes the behaviors of a culture of cells and demonstrated that the single cell approach does not account for the effects of population heterogeneity and is therefore at risk of producing erroneous results. For this application, we demonstrated that bistability is neither necessary nor sufficient for bimodal distribution.

In incorporating stochastic effects, we have relied on a continuous description of the intracellular variables by SDE. As the stochasticity arises from the randomness of chemical transformations of a small number of reacting molecules, the variables are essentially discrete. The SSA uses the chemical Master equation which is based on discrete variables, whereas the SDE approach has found various justifications in the literature. For example, van Kampen [8] uses system size expansion to obtain continuous descriptions of the stochastic variables. Although the continuous description is known to be appropriate for relatively larger number of molecules, publications exist in the literature that demonstrate the usefulness of continuous description of discrete variables for as low as even ten particles [43]. Estimates of the expressed protein level in the system of interest here range in the thousands in the on state and roughly in the range 14–35 particles per cell in the off state. Arguments for these estimates are included in the Text S1.

Hence the adoption of SDE may be regarded as appropriate for this application. In addition, the analysis of populations involves several cells of small variations about a given state so that intracellular behavior averaged among them qualifies for the SDE approach even more than in an isolated single cell.

In the section of resolving bistability versus bimodal distribution, if cells act independently from each other, bimodal distribution can be observed. However, cells change distribution from bimodal to unimodal due to population effect. In general, planktonic cells diffuse freely in the culture and such an isolated situation is hardly reached, but for a cell immobilized by extracellular matrix, such as biofilms [44], an isolated situation may be possible [45]. This study simulates the response of *E. faecalis* donor cells, harboring plasmid pCF10, to pheromone concentration. At low concentrations of pheromone as found in the natural situation [46], [47], for a perfectly mixed system all cells are predicted to be at off-state as shown in Figure 5**B**. However, for unmixed systems, non-uniformity of inhibitor concentration can lead to some cells being at the off-state, others at the on-state which together make up a bimodal distribution for the integrated population. This provides a possible mechanism for the observation of bimodal behaviors under naturally occurring conditions such as biofilms involved in dissemination of antibiotic resistance as has been shown in recent work (Cook 2010, unpublished work). Our effort in this paper has been to show that a cell in a population can behave in a significantly different manner as its environment is altered by the concerted action of other cells. A natural follow-up to this paper is the modeling of the transfer of drug resistance accounting for the presence of donor and recipient cells in different environments for which population balances will indeed provide the proper framework.

## Supporting Information

Text S1 Supporting information includes the proof of how bistability is excluded from pCF10 system, qualitative consistency between model predictions of pCF10 system and experimental observation, and the estimation of protein number per cell.
(DOC)
