# Correction: Bistability versus Bimodal Distributions in Gene Regulatory Processes from Population Balance

**DOI:** 10.1371/annotation/9c1b51d2-1957-43ff-81bc-da833eda0b8c

**Published:** 2011-10-11

**Authors:** Che-Chi Shu, Anushree Chatterjee, Gary Dunny, Wei-Shou Hu, Doraiswami Ramkrishna

In the Funding section, the NIH grant number contained an error. The correct grant number is: R01GM081388.

