# Supplementary material for: Bistability versus Bimodal Distributions in Gene Regulatory Processes from Population Balance
Source: PLoS Comput Biol. 2011 Aug 25;7(8):e1002140. doi: 10.1371/journal.pcbi.1002140 (PMC3161895; doi:10.1371/journal.pcbi.1002140)
Supplement: Text S1 — Supporting information includes the proof of how bistability is excluded from pCF10 system, qualitative consistency between model predictions of pCF10 system and experimental observation, and the estimation of protein number per cell. (DOC) [file pcbi.1002140.s001.doc]

Text S1

**How are Bistability and Bimodal Distribution of Protein Expression related in Gene Regulation? The Answer Lies in Population Balance**

Che-Chi Shu, Anushree Chatterjee, Gary Dunny, Wei-Shou Hu, and Doraiswami Ramkrishna

**Excluding the possibility of bistability**

For pheromone induced conjugation of pCF10, bistability of b (PrgB protein) with respect to c (pheromone) with the range of parameter values shown in Table 2. This bistability can be (analytically) excluded by forcing parameter$K_{4,2}$, the degradation rate of Q_s_ mRNA, equal to $K_{4,1}$, the degradation rate of Q_L_ mRNA. To get this result, we first substitute $K_{4,2}=K_{4,1}$into Eq. (2) of main text, and sum it with Eq (1) of main text. We have

| $\frac{d(q_{s}+q_{L})}{dt}=k\cdot[K_{1,1}o+K_{1,2}(1-o)]-(K_{4,1}+u)\cdot(q_{s}+q_{L})$ |  |
| --- | --- |

Then applying steady state to Eq. of Text S1 and Eqs. (3)-(7) of main text, we have

| $o=\frac{i^{r}}{i^{r}+K_{3,8}\cdot c^{r}}$ $(q_{s}+q_{L})=k\cdot[K_{1,1}o+K_{1,2}(1-o)]/(K_{4,1}+u)$ $q_{L}=\{k\cdot[K_{1,1}o+K_{1,2}(1-o)]\cdot(\frac{1}{1+K_{3,5}\cdot q_{a}})\}/(K_{4,2}+u)$ $\begin{matrix} q_{a}=\frac{\sqrt{(\beta^{2}+4\cdot(K_{4,3}+u)\cdot[K_{1,1}o+K_{1,2}(1-o)]\cdot K_{3,5})}-\beta}{2\cdot(K_{4,3}+u)} \\ where:\beta=\{[K_{1,1}o+K_{1,2}(1-o)]-[K_{1,3}o+K_{1,4}(1-o)]\}\cdot K_{3,5} \end{matrix}$ $i=K_{2,6}I/(K_{4,6}+u)$ $b=\{K_{1,7}+\frac{K_{1,9}}{1+\exp[-K_{3,9}\cdot(q_{L}-K_{1,8})]}\}/(K_{4,9}+u)$ or $b=K_{1,5}q_{L}/(K_{4,9}+u)$ $I=K_{1,6}(q_{s}+q_{L})/(K_{2,6}+K_{4,5}+u)$ |  |
| --- | --- |

The solutions of Eq. in Text S1 are the steady state values of the system. If there is only one solution, system has unique steady state. If there are multiple solutions, the system has bifurcation. To examine it, because the Eqs. are not all coupled we can separate them into Eqs. and in Text S1.

| $o=\frac{i^{r}}{i^{r}+K_{3,8}\cdot c^{r}}$ $(q_{s}+q_{L})=k\cdot[K_{1,1}o+K_{1,2}(1-o)]/(K_{4,1}+u)$ $I=K_{1,6}(q_{s}+q_{L})/(K_{2,6}+K_{4,5}+u)$ $i=K_{2,6}I/(K_{4,6}+u)$ |  |
| --- | --- |

and

| $\begin{matrix} q_{a}=\frac{\sqrt{(\beta^{2}+4\cdot(K_{4,3}+u)\cdot[K_{1,1}o+K_{1,2}(1-o)]\cdot K_{3,5})}-\beta}{2\cdot(K_{4,3}+u)} \\ where:\beta=\{[K_{1,1}o+K_{1,2}(1-o)]-[K_{1,3}o+K_{1,4}(1-o)]\}\cdot K_{3,5} \end{matrix}$ $q_{L}=\{k\cdot[K_{1,1}o+K_{1,2}(1-o)]\cdot(\frac{1}{1+K_{3,5}\cdot q_{a}})\}/(K_{4,2}+u)$ $b=\{K_{1,7}+\frac{K_{1,9}}{1+\exp[-K_{3,9}\cdot(q_{L}-K_{1,8})]}\}/(K_{4,9}+u)$ or $b=K_{1,5}q_{L}/(K_{4,9}+u)$ |  |
| --- | --- |

To see the number of solutions of b with respect to each value of c, we first start with in Text S1. Clearly, $b$can be written as a function of o (DNA in looped form). $b$is unique if o is unique in Eq. in Text S1 with respect to each value of c. To examine it, we further rewrite Eq. in Text S1 as Eqs. and in Text S1

| $i^{r}=\frac{o\cdot K_{3,8}\cdot c^{r}}{(1-o)}$ |  |
| --- | --- |
| $i=(K_{1,1}-K_{1,2})m\cdot o+K_{1,2}m$ |  |
| $where: m=K_{2,6}K_{1,6}k\cdot/[(K_{4,1}+u)\cdot(K_{2,6}+K_{4,5}+u)\cdot(K_{4,6}+u)]$ |  |

An efficient way to analyze the number of solutions of Eqs. and in Text S1 is to plot these two, an example is shown in figure S1. For Eq. in Text S1, $i$ is a monotonic function with respect to $o$ because the concentration of intracellular inhibitor, $i$, cannot be negative or complex. For Eq. in Text S1, $m$ is a positive constant, so that the slope is decided by $(K_{1,1}-K_{1,2})$. Because $K_{1,1}$ is the transcription rate of DNA in the looped (repressed) form and $K_{1,2}$ is the transcription rate of DNA in the unlooped (active) form, $(K_{1,1}-K_{1,2})$is always negative. Therefore we conclude that $b$ is unique for each value of c. Bistability of system is thus excluded.

Figure S1 The plot of intracellular inhibitor, $i$, with respect to DNA in repressed form, $o$. The curve is obtained from Eq. in Text S1 and line is obtained from Eq. in Text S1. The parameter values used here are as in Table 2 except $K_{4,2}=K_{4,1}$ and extracellular pheromone concentration is 0.88nM. This plot illustrates the idea that only one intersection between the curve and the line is possible.

**Qualitative consistency between simulation and literature observation**

The figure S2 a) is figure 3 (B) in the work of Hirt et. al. (2005). Pheromone cCF10 is added at the beginning and, at certain time points, RNA is measured by real-time PCR. The figure S2 b) and c) are simulation results, which qualitatively show consistent with observations in the literature, using Eq. (1)-(4) and (6)-(8) of main text.

a)

b)

c)


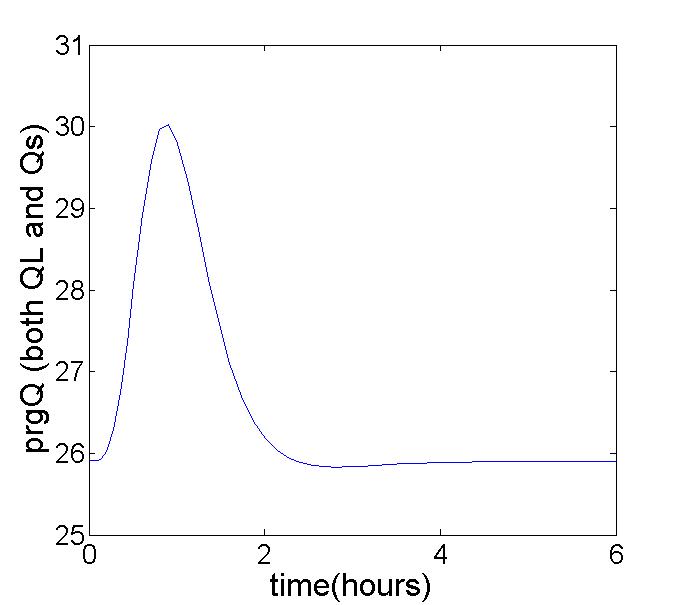

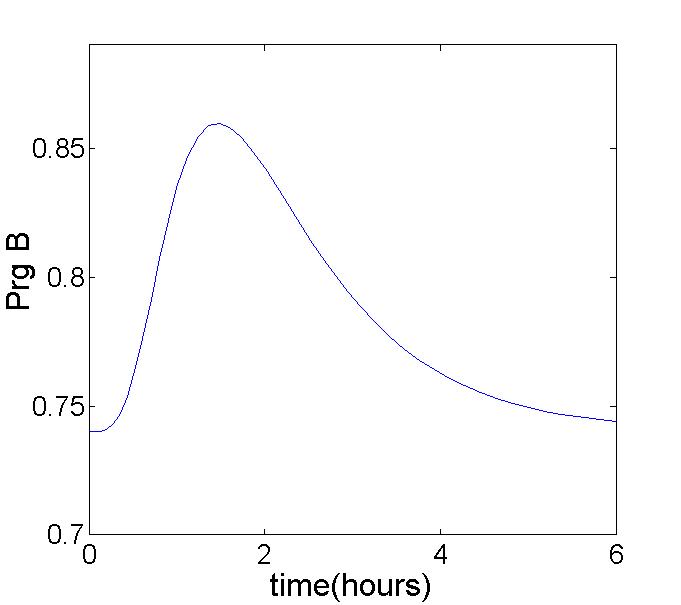


Figure S2 Qualitative consistency between simulation and literature observation. a) is the literature observation done by Hirt et. al. (2005) as figure 3 (B) in their work. b) and c) are simulation results which are qualitatively consistent with experimental observations for both prgQ and PrgB.

**The estimation of protein number per cell**

There is no measure of number of PrgB molecules on surface of the cell, but we do have an estimation of RNA levels for both *prgX* and *prgB* transcript. Based on unpublished data (by Dawn Manias) the number of PrgX molecules inside pCF10 carrying donor cell ranges between 700-2000 molecules per cell. This is based in Quantitative Western Blot and Quantitative PCR. On an average under uninduced conditions *prgX* mRNA is 20-50 times more than prgB mRNA (based on QPCR), whereas in the induced state *prgB* mRNA is more than *prgX* mRNA. If we assuming that the level of protein is proportional to level of RNA and that the translational efficiency of proteins PrgX and PrgB are similar, the roughly number of PrgB is 14-35 (700/50-700/20) in uninduced state.

REFERENCE

Hirt H, Manias DA, Bryan EM, Klein JR, Marklund JK, et al. (2005) Characterization of the pheromone response of the Enterococcus faecalis conjugative plasmid pCF10: Complete sequence and comparative analysis of the transcriptional and phenotypic responses of pCF10-containing cells to pheromone induction. Journal of Bacteriology 187: 1044-1054
